# Toxic Effects of Copper and Zinc Oxide Nanoparticles on Brain Tissue Antioxidant Defense of Male Swiss Albino Mice

**DOI:** 10.1007/s12011-025-04964-9

**Published:** 2026-02-06

**Authors:** Özge Temiz, Dicle Kargin

**Affiliations:** 1 Vocational School of Health Services, Osmaniye Korkut Ata University, Osmaniye, 80000 Turkey; 2 Faculty of Health Sciences, Van Yüzüncü Yıl University, Van, 65090 Turkey

**Keywords:** Copper oxide, Zinc oxide, Mice, Oxidative stress, Genotoxicity

## Abstract

The aim of this study was to evaluate the general toxicity of nanoscale copper oxide (CuO NPs) and zinc oxide nanoparticles (ZnO NPs) by oral gavage in mice. In the study conducted with 42 male Swiss albino mice, CuO NPs and ZnO NPs were administered at 3 different doses of 1, 5 and 25 mg/kg/day by oral gavage method for 14 days together with the control group (*n* = 6). GSH and GSH dependent enzymes GST, GPx and GR enzyme activities as well as thiobarbituric acid reagent (TBARS) levels, DNA oxidative damage (8-Hydroxy-2’-deoxyguanosine; 8OHdG) and oxidative protein damage (protein carbonyl; PC) were determined in male mice brain tissue after 14 days of exposure to different doses of CuO NPs and ZnO NPs by spectrophotometric methods. There was a decrease in GSH levels and a dose-dependent increase in GST, GPx and GR enzymes in the brain tissue of male mice at CuO NPs and ZnO NPs doses. In the brain tissue of male mice exposed to CuO NPs and ZnO NPs doses for 14 days, TBARS, 8-OHdG and PC levels increased compared to the control group. According to these findings, CuO NPs and ZnO NPs were shown to have dose-dependent toxic effects, inducing oxidative damage by inducing the antioxidant system in the brain of mice, and also causing negative effects on the physiological basic structures of biomolecules such as DNA, fat and protein. Our results show that CuO nanoparticles have a more toxic effect on brain tissue than ZnO nanoparticles, therefore, neurotoxic effects were determined in a dose-dependent manner and in relation to the examined antioxidant parameters.

## Introduction

Increasing use of nanomaterials, heavy metals that threaten living organisms in the entire ecological system, including humanity, cause environmental pollution [1]. According to the biological functional role of heavy metals in living things, heavy metals are classified as essential and non-essential [2, 3]. Heavy metals, which are in the basic group in biological processes, are necessary for living organisms such as zinc and copper [4]. With these properties, especially CuO and ZnO NPs are often found in food supplements for humans. Its unique physicochemical properties participate in functional applications in physiological metabolic processes and as a result increase their commercial value in the health (cosmetic, supplement) industry [5–8]. Dietary zinc intake is important for human health and beneficial for disease treatment [9]. The human body can only absorb 20–40% of the zinc ingested through food. ZnO NPs have replaced the additive ZnO to increase zinc uptake in the food industry [10]. Additionally, copper is an important element for growth and development in organisms. Copper plays a role as a cofactor for enzymes involved in various biological reactions such as photosynthesis, respiration, free radical detoxification. With the use of copper NPs for human health; they are used in many areas as antibacterial products, anti-tumor and osteoporosis treatment drugs, and as an additive in the feed of farm and poultry, which is a food product, and as a pesticide in agricultural products [11–13]. In vivo and in vitro studies have shown that uptake of copper and zinc nanoparticles can induce biological effects and induce oxidative stress [14–18]. Developing oxidative stress as a consequences of the nanoparticles’ dispersion within the cell, and because of their unique properties, the bioeffects of nanoparticles may interact differently when used alone or in combination [14, 18]. As a result of aerobic metabolism activities, it produces reactive oxygen species (ROS) with unpaired electrons under physiological conditions. Oxidative stress represents an imbalance between the generation of ROS and the ability of the aerobic system to detoxify reactive intermediates or to repair the damage caused by the resulting excess ROS [15]. Changes in the levels of ROS are tried to be kept in balance with a few parameters. These are antioxidant enzymes, alteration of mitochondrial electron transfer chain, fenton reaction or levels of cellular glutathione (GSH) [19].

In case of insufficient defense mechanisms, especially in the metabolism of metal ions, oxidative stress formation can induce protein carbonylation (PC), lipid peroxidation (LPO), DNA damage and apoptosis [20–22]. The aim of this study is to examine the effects of trace elements on brain functions on antioxidant system and various biochemical pathways.

## Materials and Methods

### Chemicals

CuO NP nanopowder, CAS Number: 1317-38-0 ∼40 nm, ZnO NP nanopowder, CAS Number: 1314-13-2∼40 nm were obtained from Sigma-Aldrich (Germany). All analytical grade chemicals and reagents, including Bradford reagent, glacial metaphosphoric acid, Disodium EDTA, 5,5’-dithio-bi[2-nitrobenzoic acid] (DTNB), Tris Base, t-butyl hydroperoxide, Sodium Carbonate, NADPH, GR, GSH, 1-chloro-2,4-dinitrobenzene (CDNB), Sodium dodecyl sulfate, Acetic acid, Thiobarbituric acid, n-Butanol, pyridine, 2,4-dinitrophenylhydrazine (DNPH), Trichloroacetic Acid, Sodium Dihydrogen Phosphate, were purchased from Merck and Sigma Chemical Co. (St Louis, MO, USA). Additionally, the Mouse 8-OHdG (8-Hydroxydeoxyguanosine) ELISA Kit used in the kit study was purchased from Fine Test Company.

### Animals

The total number of mice used in our study (*N* = 42) was determined by power analysis using the G*Power Program (version 3.1.9.7; Heinrich-Heine University, Düsseldorf, Germany) [23]. For use in toxicology studies: 42 male Swiss albino mice (*Mus musculus*), 9–10 weeks old, 26–30 gr in weight, were obtained from the Çukurova University Health Sciences Experimental Application and Research Center and were taken to the experimental environment in this center. The mice were housed in stainless steel cages in a continuously ventilated animal room. Control and experimental mice were provided with normal pellet feed and unlimited access to drinkable tap water ad libitum under the same conditions. The experimental animals were cared for under appropriate conditions according to approved procedures in an environment where physiological conditions were provided, with room temperature of 23 ± 2 °C, humidity of 55 ± 4% and a 12-hour day/night period to provide a circadian period during the day. An ethics committee report was received from the Local Ethics Committee (SABİDAM) of the Health Sciences Experimental Application and Research Center at Çukurova University, which allows animal experiments for research purposes with the decision numbered 20/1/5.

### Characterization of NPs

Scanning electron microscope images were analyzed to determine the characteristic features of NPs, to determine the metal size and compare with the manufacturer’s data and described in detail in the previous study [24] Before each treatment, stock solutions of CuO-NPs and ZnO-NPs were sonicated (Bandelin HD2200 brand, Germany) for 20 min before oral gavage.

### Experimental Design

For toxicity experiments, mice (*n* = 6) were randomly distributed into 7 groups in the study that lasted for 14 days. 100 µl of solution containing NPs was administered to mice via oral gavage. The tests included three sublethal NP dose levels (1, 5 and 25 mg/kg/day) and a control group (100 µl of water for placebo). We determined the doses of our study based on nominal doses determined through preliminary trials. Determination of nanoparticle toxic levels was used in our research according to lethal dose levels determined in previous studies [25, 26]. Then, at the end of the experimental process, mice were sacrificed under general anesthesia with intraperitoneal injection of ketamine and xylazine at a rate of 20/1 ppm. Brain tissues were removed from anesthetized mice and stored at −80 °C until biochemical parameters were studied.

### Biochemical Analysis

Brain tissues were divided into 2 parts for the protocols of biochemical parameters. In the first part, the tissues were homogenized in PBS buffer containing 2.5 mM ATP with pH 7.4 in a ratio of 1/10 (w/v) for brain tissue. The supernatants were obtained by centrifugation at 16000×g for 20 min. at 4 °C. The antioksidant system parameters were determined from supernatants obtained from brain tissues of male mice using protein level was determined using the Bradford (1976) method [27], TBARS level using the Ohkawa et al. (1979) method [28], GSH level using the Beutler (1975) method [29] and PC level using the Levine et al. (1990) [30] method. The enzyme activities were determined from supernatants obtained from brain tissues of male mice using the GST Habig et al. (1974) method [31], GPx Beutler (1984) method [32] and GR Carlberg and Mannervik, (1975) method [33]. DNA oxidation biomarker 8-OHdG levels commercial (kit procedures using SUNRED 8-OHdG levels uses the Competitive-ELISA principle ELISA kit method were determined from the second part brain tissues.

### Statistical Analysis

Statistical analyses of biochemical analysis data were performed using the one-way Anova-Duncan test (*P* < 0.05) and Levene’s tests to confirm statistical normality. These analyses were performed using the SPSS 22.0 (SPSS Inc., Chicago, IL) package program.

## Results

**Fig. 1:**
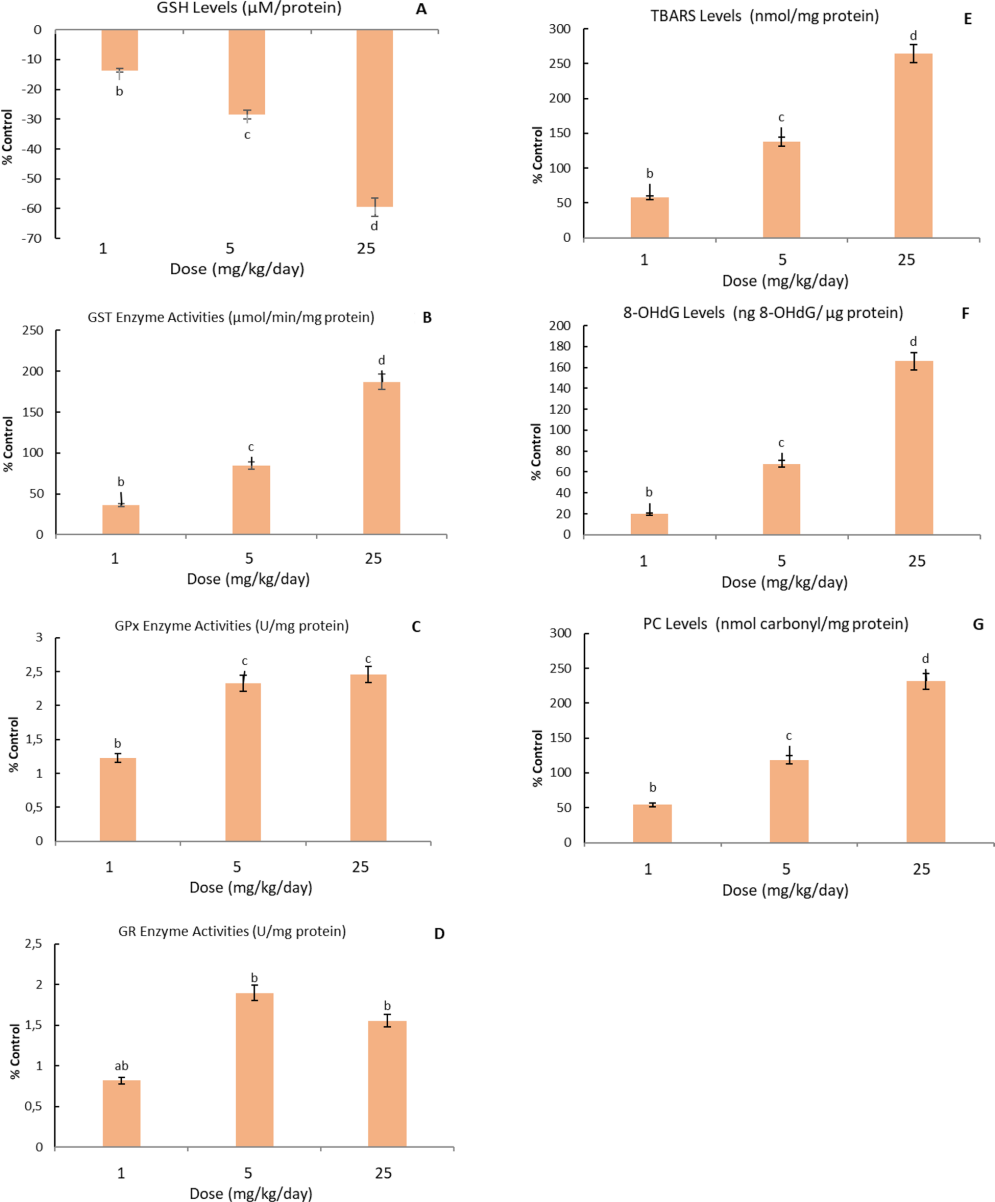
In the brain tissue of male mice exposed to CuO NPs (0, 1, 5 and 25 mg/kg-day) orally for 14 days, changes in the levels of GSH (**A**), GSH-dependent enzymes GST (**B**), GPx (**C**) and GR (**D**) enzyme activities were given compared to the control group. Data show the mean values and associated standard deviation (SD) of 6 male mice. Data shown with different letters in the figures indicate a significant difference between the control group and CuO NPs exposure (P < 0.05). In the brain tissue of male mice exposed to CuO NPs (0, 1, 5 and 25 mg/kg-day) orally for 14 days, changes in the levels of TBARS (**E**), 8-OHdG (**F**) and PC (**G**) were given compared to the control group. Data show the mean values and associated standard deviation (SD) of 6 male mice. Data shown with different letters in the figures indicate a significant difference between the control group and CuO NPs exposure (P < 0.05)

**Fig. 2:**
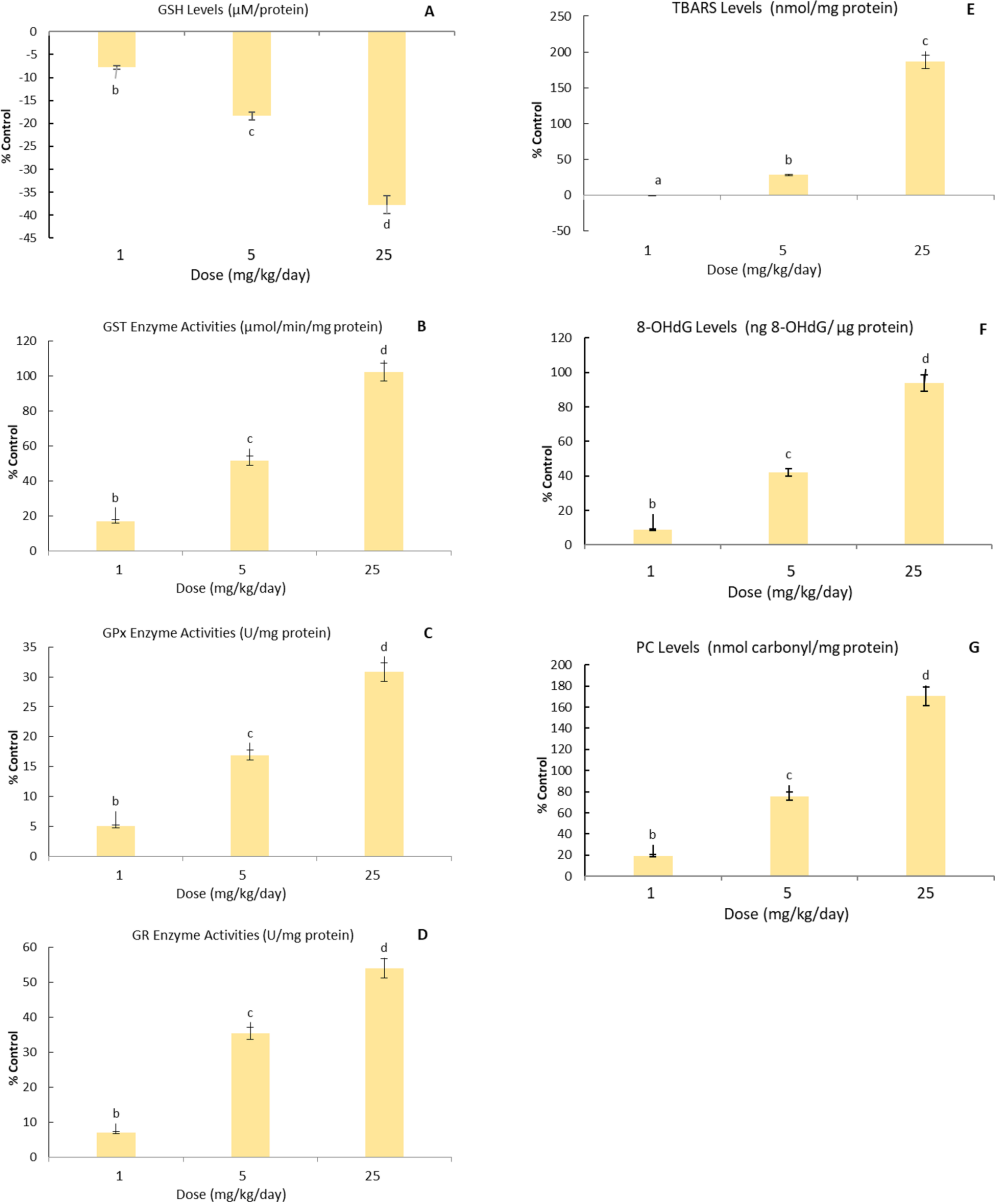
In the brain tissue of male mice exposed to ZnO NPs (0, 1, 5 and 25 mg/kg-day) orally for 14 days, changes in the levels of GSH (**A**), GSH-dependent enzymes GST (**B**), GPx (**C**) and GR (**D**) enzyme activities were given compared to the control group. Data show the mean values and associated standard deviation (SD) of 6 male mice. Data shown with different letters in the figures indicate a significant difference between the control group and ZnO NPs exposure (P < 0.05). In the brain tissue of male mice exposed to ZnO NPs (0, 1, 5 and 25 mg/kg-day) orally for 14 days, changes in the levels of TBARS (**E**), 8-OHdG (**F**) and PC (**G**) were given compared to the control group. Data show the mean values and associated standard deviation (SD) of 6 male mice. Data shown with different letters in the figures indicate a significant difference between the control group and ZnO NPs exposure (P < 0.05)

The dose-dependent increase and decrease in GSH level, GST, GPx, GR enzyme activities and TBARS, 8-OHdG and PC levels in mice brain tissue with the application of CuO NPs doses are indicated in Figs. 1A, 1B, 1C, 1D, 1E, 1 F and 1G, respectively, and were evaluated against the control group. Dose-dependent increases and decreases in GSH levels, GST, GPx, GR enzyme activities, and TBARS, 8-OHdG, and PC levels in mouse brain tissue with the application of ZnO NPs doses are shown in Figs. 2A, 2B, 2C, 2D, 2E, 2F, and 2G, respectively, and were evaluated by comparing with the control group.

### The Changes in GSH Levels and GSH-Dependent Enzyme Activities

In the effect of 14-day exposure to CuO NPs doses of 1, 5 and 25 mg/kg/day, it was determined that there was a dose-dependent decrease in GSH levels in mice brain tissue by 14%, 29% and 60%, respectively. Percentage change rates and statistical differences compared to the control group are shown in Fig. 1 A, *p* < 0.05.In the effect of 14-day exposure to CuO NPs doses of 1, 5 and 25 mg/kg/day, it was determined that there was a dose-dependent increase in GST enzyme activity in mice brain tissue by 36%, 85% and 187%, respectively. Percentage change rates and statistical differences in the contrary to the control group are shown in Fig. 1B, *p* < 0.05. At the end of 14 days of exposure to CuO NPs doses of 1, 5 and 25 mg/kg/day, a dose-dependent increase in GPx enzyme activity was determined in the brain tissue of mice by 2%, 3% and 3%, respectively. Percentage change rates and statistical differences in the contrary to the control group are shown in Fig. 1 C, *p* < 0.05. In the effect of 14-day exposure to CuO NPs doses of 1, 5 and 25 mg/kg/day, it was determined that there was a dose-dependent increase in GR enzyme activity in mice brain tissue by 1%, 2% and 2%, respectively. Percentage change rates and statistical differences in the contrary to the control group are shown in Fig. 1D, *p* < 0.05.

Following 14-day exposure to 1, 5, and 25 mg/kg/day ZnO NPs, a dose-dependent decrease in GSH levels in the brain tissue of mice was determined by 8%, 17%, and 37%, respectively. Percentage change rates and statistical differences in the contrary to the control group are shown in Fig. 2 A, *p* < 0.05. Following 14-day exposure to 1, 5 and 25 mg/kg/day ZnO NPs, a dose-dependent increase in GST enzyme activity in the brain tissue of mice was determined by 17%, 52% and 102%, respectively. Percentage change rates and statistical differences in the contrary to the control group are shown in Fig. 2B, *p* < 0.05. Following 14-day exposure to 1, 5, and 25 mg/kg/day ZnO NPs, a dose-dependent increase in GPx enzyme activity in the brain tissue of mice was determined by 5%, 17%, and 31%, respectively. Percentage change rates and statistical differences in the contrary to the control group are shown in Fig. 2 C, *p* < 0.05. Following 14-day exposure to 1, 5, and 25 mg/kg/day ZnO NPs, a dose-dependent increase in GR enzyme activity in the brain tissue of mice was determined by 7%, 36%, and 54%, respectively. Percentage change rates and statistical differences in the contrary to the control group are shown in Fig. 2D, *p* < 0.05.

### TBARS Level

In the effect of 14-day exposure to CuO NPs doses of 1, 5 and 25 mg/kg/day, it was determined that there was a dose-dependent increase in TBARS level in mice brain tissue by 58%, 138% and 264%, respectively. Percentage change rates and statistical differences in the contrary to the control group are shown in Fig. 1E, *p* < 0.05. In the effect of 14-day exposure to ZnO NPs doses, there was no change in TBARS levels in the brain tissue of mice at 1 mg/kg/day dose, respectively. A dose-dependent increase of 28% and 186% in TBARS levels was determined at 5 and 25 mg/kg/day ZnO NPs doses. Percentage change rates and statistical differences compared to the control group are shown in Fig. 2E, *p* < 0.05.

### 8-OHdG Levels

In the effect of 14-day exposure to CuO NPs doses of 1, 5 and 25 mg/kg/day, it was determined that there was a dose-dependent increase in 8-OHdG level in mice brain tissue by 20%, 68% and 166%, respectively. Percentage change rates and statistical differences compared to the control group are shown in Fig. 1 F, *p* < 0.05. In the effect of 14-day exposure to ZnO NPs doses of 1, 5 and 25 mg/kg/day, it was determined that there was a dose-dependent increase in 8-OHdG level in mice brain tissue by 8%, 42% and 94%, respectively. Percentage change rates and statistical differences compared to the control group are shown in Fig. 2 F, *p* < 0.05.

### PC Levels

In the effect of 14-day exposure to CuO NPs doses of 1, 5 and 25 mg/kg/day, it was determined that there was a dose-dependent increase in PC level in mice brain tissue by 54%, 118% and 251%, respectively. Percentage change rates and statistical differences in the contrary to the control group are shown in Fig. 1G, *p* < 0.05. Following 14-day exposure to 1, 5, and 25 mg/kg/day ZnO NPs, a dose-dependent increase in PC levels in the brain tissue of mice was determined by 20%, 76%, and 170%, respectively. Percentage change rates and statistical differences in the contrary to the control group are shown in Fig. 2G, *p* < 0.05.

## Discussion

NPs exhibit quite different toxicological effects compared to products with the same chemical content but larger sizes. The factors that affect the toxicity of all chemicals are generally the same basic factors that affect the toxicity of CuO NPs and ZnO NPs. These are various biochemical and physical properties such as size, shape, and concentration [34]. NPs can easily enter the cell with the advantage of their small size and can produce ROS, affecting normal cellular functions. Excessive ROS formation causes oxidative stress in the cell and results in a decrease in GSH levels due to the depletion of the antioxidant system element reduced glutathione (γ-glutamyl-cysteinyl-glycine, tripeptide) as a result of ROS detoxification [35].

In our study, the decrease in GSH levels in the brain of mice in CuO NPs and ZnO NPs applications depending on the dose can be interpreted as the antioxidant system is triggered against the toxicity and the defense mechanism is activated to protect the cell. Intracellular GSH levels are an important factor in determining the antioxidant system defense mechanism that the cell has and are important together with the enzymes that regulate the amount of cellular GSH. In another study, according to parallel results, it was reported that intraperitoneal applications of cerium oxide nanoparticles at 50 µg/kg/bw and zinc oxide nanoparticles at 80 µg/kg/bw to male wistar rats three times a week for 4 weeks resulted in a decrease in GSH levels in liver and kidney tissues in the contrary to the control group [36]. In their study, Abdelazeim et al. (2020) [37] reported a significant decrease in GSH levels in rat liver tissue after 2 weeks of daily application of CuO NPs at 100 mg/kg compared to the control group. Yahya et al. (2019) [38] studies reported a decrease in liver GSH levels following oral administration of ZnO NPs (10 mg/kg/day) and intraperitoneal injection of CuO NPs (0.5 mg/kg/day) to rats for 28 days. GST enzyme activity was reported to increase compared to control in both nanoparticle applications. GSH and GSH dependent enzymes glutathione-s-transferase (GST), glutathione peroxidase (GPx) and glutathione reductase (GR) enzyme activities play a role in cellular protection against oxidative stress conditions. Interaction with sulfhydryl (SH) groups in the GSH structure is usually provided by the dissolution of metal ions in the nanoparticles. Thus, metals bound to thiols inactivate GSH, a low molecular weight antioxidant, and allow the conjugation of metals, which are toxic, with GST enzyme activity [39, 40].

In our study, an increase in GST activity was observed in mice brain tissue in CuO and ZnO NPs doses depending on the dose in the contrary to the control group. Although there was a statistical difference in GPx and GR enzymes depending on the dose, no significant percentage change was determined compared to the control, especially in 5 and 25 mg/kg/day applications. These changes in GSH-dependent enzymes in the study reveal that CuO NPs and ZnO NPs were attempted to be detoxified through GSH conjugation via electron binding, and that GPx and GR enzyme activities were not sufficiently active, which is why they were not active enough. These enzymes are important structures that are responsible for maintaining and regulating cellular GSH levels. The GR enzyme activity acts as a hydrogen donor and carries out the reaction of converting oxidized GSH (GSSG) to GSH using nicotinamide adenine dinucleotide phosphate (NADPH) [41, 42]. The GPx enzyme removes cellular peroxides by using GSH as a substrate during detoxification, while the GST enzyme catalyzes the binding of electrophilic compounds to cellular GSH, thus preventing cell damage by ensuring the excretion of toxins [43]. A different result from our study was found in the Shrivastava et al. (2014) [44] study, in which an oral dose of 500 mg/kg/day ZnO NPs was used for 21 days, a decrease in mouse liver GST and GPx enzyme activities, an increase in brain tissue GST and a decrease in GPx enzyme activities were determined in the contrary to the control group. In addition, an increase in GST and GPx enzyme activities was reported in blood erythrocyte cells. These results show that nanoparticles can provide dose, time and tissue specific responses in living organisms. In the study conducted by Singh et al. (2020) [45] GPx enzyme activity in liver tissues of rats exposed to 50 and 250 mg/kg ZnO NP showed a decrease with increasing ZnO NP dose. They showed that GR enzyme activity decreased in the high dose effect compared to the control group. Liu et al. (2014) [46] indicated in their study that there was no alter in GST and GPx activities in brain tissue after exposure to 40 mg/kg/day high-dose CuO NP via inhalation. In in vitro studies by Fahmy and Cormier (2009) [47], it was determined that exposure to copper oxide nanoparticles at 400 µg/cm^2^ on human laryngeal epithelial cells resulted in inhibition of GR enzyme activity and increase in GPx enzyme activity compared to cells exposed to only the nutrient.

According to the results determined in the studies, it has been shown that free radicals also interact with lipids, proteins and DNA in the cell wall and structural activities of cell biomolecules, and reveal oxidized end products associated with mutagenesis and apoptosis [20, 48, 49]. The increase in ROS, which attack structural lipids in the cell membrane, occurs due to the increase in TBARS levels and increased tissue lipid peroxidation [50]. In the investigation we conducted, an increase in brain tissue TBARS levels was observed in all doses under the effect of CuO and ZnO NP, depending on the dose, compared to the control. Similar results were found in the previous study by Fadoju et al. (2019) [51] where it was reported that ZnO NP exposure in the tissues of swiss albino mice increased TBARS levels compared to control at different times and doses. Many studies have reported an increase in TBARS levels in the tissues of mice and rats exposed to CuO NP and ZnO NP at various doses and durations compared to controls [52–55].

ROS can react with cell nucleophilic structures as a result of toxicity and bind covalently to DNA molecules, resulting in single-strand breaks, double-strand breaks, and the formation of DNA adducts, which can lead to DNA damage. ROS attack on DNA can cause a wide variety of base and sugar modifications on DNA strands. DNA cleavage, DNA-protein crosslinks, and oxidation of purines are seen in DNA, especially in reactions with ROS and hydroxyl radicals. Oxidized bases such as 8-Oxo-7,8-dihydro-2′-deoxyguanosine (8-oxodGuo; also called 8-OHdG) change their base pairing properties with adenine instead of cytosine, potentially causing mutagenic interactions [35, 56, 57]. In our study, an increase in brain tissue 8-OHdG levels under the effect of CuO and ZnO NP was observed at all doses, depending on the dose, compared to the control. In the previous study by Attia et al. (2018) [55], it was observed that there was DNA fragmentation and DNA damage as a result of DNA comet analysis data at the end of the effect of 40 and 100 mg/kg/day doses of ZnO NPs for 7 days. Attia et al. (2021) [49] reported in their study that CuO NP doses caused an increase in lipid peroxidation marker TBARS levels and massive DNA fragmentations as a result of DNA analysis. In study conducted by Sharma et al. (2012) [52], it was determined that there was an increase in TBARS levels in the liver and kidney tissues of mice exposed to ZnO NP 50 and 300 mg/kg/day doses for 14 days, and there was no statistically significant increase in brain tissue compared to the control group. In addition, a significant increase was found in liver and kidney cells in the Fpg-modified Comet assay at a dose of 300 mg/kg/day compared to the control group for oxidative DNA damage. However, no significant DNA damage was observed in mice administered 50 mg/kg/day doses of ZnO NP. It was reported that no changes were detected in the kidney tissue of mice exposed to ZnO NP compared to the control group. Many studies have reported that exposure to CuO NP and ZnO NP causes an increase in the levels of biomolecular parameters TBARS and oxidative DNA damage, and the toxic effect increases with increasing dose [58–60].

The formation of protein carbonyls as a result of oxidative stress in proteins, which have an important place in biomolecular structures, is the final product as an oxidative protein lesion [61, 62]. Carbonylation formation is irreversible and usually a degeneration in the chains of the protein structure, formation of structures such as misfolding, breakage, aggregation, causes the protein function to deteriorate and fail to perform its functions [63]. Protein carbonyls formed as a result of oxidative damage in proteins with different amino acid sequences appear as aldehydes and ketones. This is due to damage caused by the fragmentation of the peptide chain in a different order, oxidation as a side chain, or a secondary reaction by interaction with oxidized cellular metabolites [61, 64, 65]. In the previous study by Majewski et al. (2020) [54], it was pointed out that there was an increase in PC levels in rat blood under the effect of CuO NP compared to the control group. Exposure to oxide nanoparticles causes damage by attacking the protein structures in the cell structure with ionic metals such as Cu and Zn, which have a prooxidative effect. In their previous study by Manickam et al. (2017) [66], it was reported that there was an increase in PC levels compared to the control under the influence of a different metal oxide, Fe_2_O_3_ NPs, at doses of 25 and 50 mg/kg for 30 days.

## Conclusion

The use of CuONP and ZnO NPs in various areas raises toxicological concerns for the environment and humans. Our current study reveals the ability of these NPs to cause significant biochemical and physiological changes and oxidative stress in mice, mammalian species. In addition, changes in DNA, TBARS, PC and antioxidant system parameters by causing excessive oxidative damage on biomolecular structures can provide an important toxicological indicator of the potential toxic effects of NPs. Therefore, it is important to control the exposure of ecology and humans to such substances and ensure that they remain within safe limits.

## Data Availability

No datasets were generated or analysed during the current study.
