# Correction to: Toxic Effects of Copper and Zinc Oxide Nanoparticles on Brain Tissue Antioxidant Defense of Male Swiss Albino Mice

**DOI:** 10.1007/s12011-026-05067-9

**Published:** 2026-04-10

**Authors:** Özge Temi̇z, Dicle Kargin

**Affiliations:** 1 Vocational School of Health Services, Osmaniye Korkut Ata University, Osmaniye, 80000 Turkey; 2 Faculty of Health Sciences, Van Yüzüncü Yıl University, Van, 65090 Turkey

**Correction to: Biological Trace Element Research**

10.1007/s12011-025-04964-9

The original version of this article contained mistakes.

In this article the author’s name Özge Temi̇z was incorrectly written as Özge Temi̇z as well as the running headers.

Also, Figs 1 and 2 should be presented as:

Fig. 1. In the brain tissue of male mice exposed to CuO NPs (0, 1, 5 and 25 mg/kg-day) orally for 14 days, changes in the levels of GSH (A), GSH-dependent enzymes GST (B), GPx (C) and GR (D) enzyme activities were given compared to the control group. Data shown with different letters in the figures indicate a significant difference between the control group and CuO NPs exposure (P < 0.05, n=6). In the brain tissue of male mice exposed to CuO NPs (0, 1, 5 and 25 mg/kg-day) orally for 14 days, changes in the levels of TBARS (E), 8-OHdG (F) and PC (G) were given compared to the control group. Data shown with different letters in the figures indicate a significant difference between the control group and CuO NPs exposure (P < 0.05, n=6).
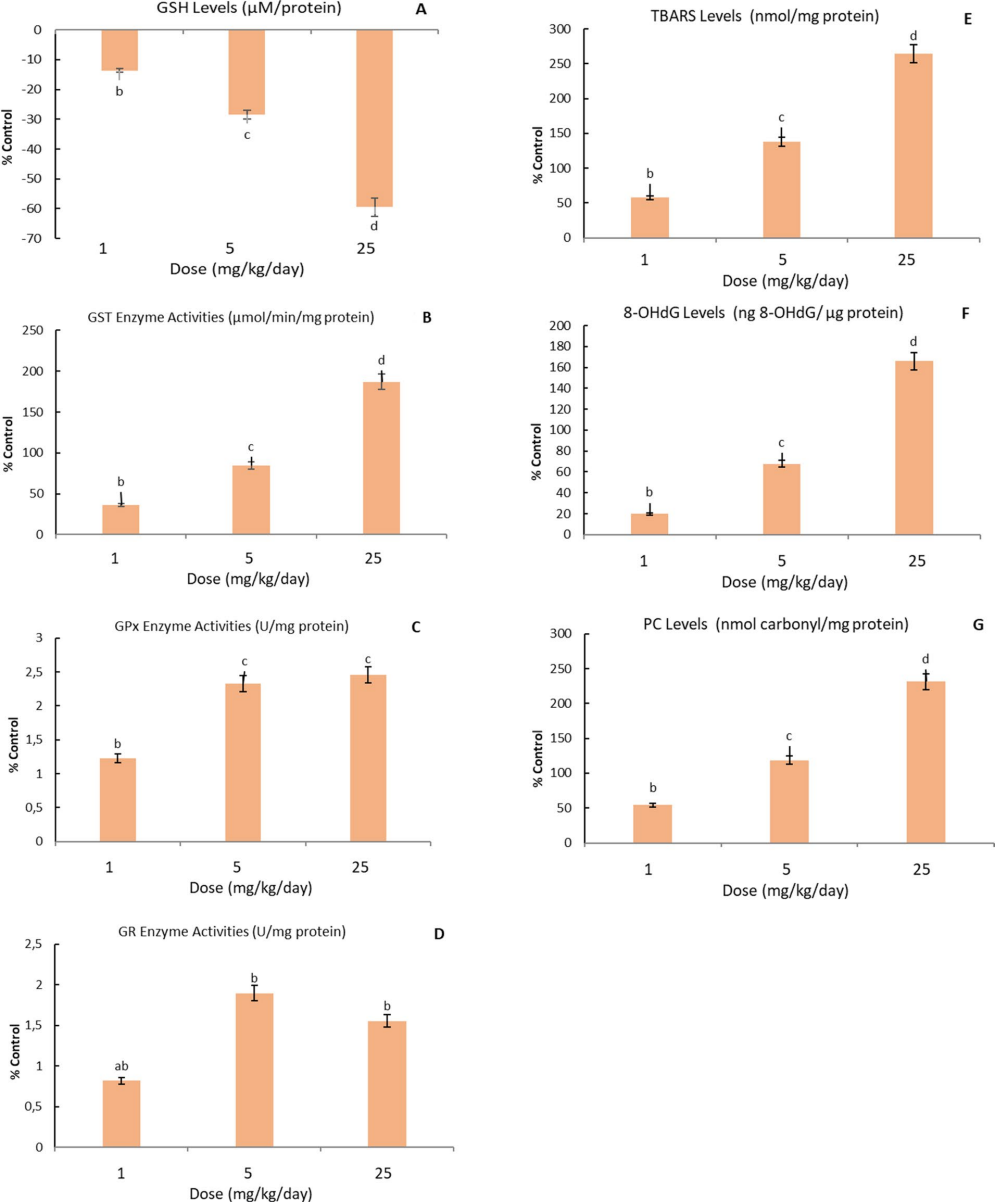


Fig. 2. In the brain tissue of male mice exposed to ZnO NPs (0, 1, 5 and 25 mg/kg-day) orally for 14 days, changes in the levels of GSH (A), GSH-dependent enzymes GST (B), GPx (C) and GR (D) enzyme activities were given compared to the control group. Data shown with different letters in the figures indicate a significant difference between the control group and ZnO NPs exposure (P < 0.05, n=6). In the brain tissue of male mice exposed to ZnO NPs (0, 1, 5 and 25 mg/kg-day) orally for 14 days, changes in the levels of TBARS (E), 8-OHdG (F) and PC (G) were given compared to the control group. Data shown with different letters in the figures indicate a significant difference between the control group and ZnO NPs exposure (P < 0.05, n=6).
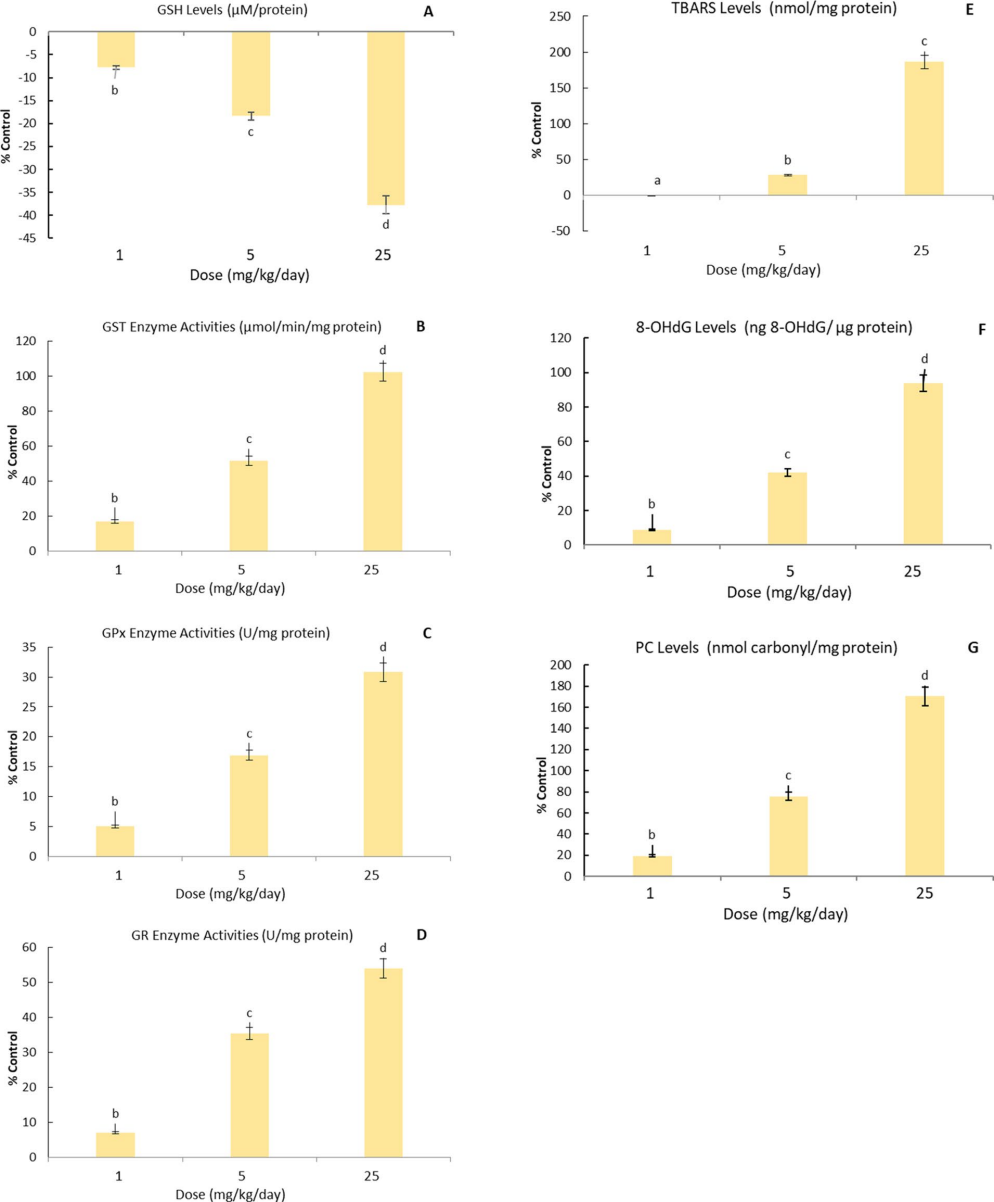


The original article has been updated.

